# “Doctors’ Lounge” podcast to teach clinical reasoning to first-year medical students

**DOI:** 10.15694/mep.2018.0000132.1

**Published:** 2018-06-14

**Authors:** Shilpa Brown, Elena Wood, Daniel McCollum, Allen Pelletier, Jennifer Rose, Paul Wallach

**Affiliations:** 1 Medical College of Georgia at Augusta University; 2 Augusta University; 3 Indiana University School of Medicine

**Keywords:** Medical students, Clinical reasoning, Flipped classrom, Physical Diagnosis

## Abstract

This article was migrated. The article was marked as recommended.

In the first year of medical school, our students have a comprehensive course in history taking, physical examination skills, clinical reasoning, and patient-centered care. We have observed that first year students struggle to conduct a focused history and perform a focused physical examination on a given chief complaint. We developed an innovative program to address this concern in our Essentials of Medicine- Physical Diagnosis course.

We created an online outline and audio podcast for students to review illustrating the key elements of the history of presenting illness, review of systems, other historical patient information, and focused physical examination for 3 specific chief complaints to assist them in their approach to these patients. This resource also included the discussion of the work up and treatment plans and was created in collaboration of Internal, Family, and Emergency Medicine to account for the various approaches to the same chief complaint within the various specialites of medicine.

Students completed a brief pre- and post-session survey to assess their utilization of the resource, quality of the content, and delivery of the session materials. The preceptor’s were also surveyed regarding the students’ ability to conduct a patient encounter and discuss their assessment and plan comparing current students to those in previous years who did not use this resource. We also asked for feedback on how these resources might be improved for future use.

The resource was highly effective for first-year medical students in preparation for focused history taking and physical examination of a patient with a specific chief complaint. Students were more engaged in the critical reasoning discussion of the case assessment and plan after using this resource and preceptors were in agreement.

We believe this model we called the “Doctors’ Lounge” developed for the chief complaints of sore throat, chest pain, and abdominal pain can be replicated at any medical school desiring to introduce or enhance teaching of clinical reasoning skills to their preclinical students.

## Introduction

The Physical Diagnosis component of the Essential Clinical Medicine module introduces students to the building blocks needed to become an excellent physician, which include core knowledge, attitudes, and skills related to interviewing and examining patients. Students learn the format and specifics of a complete history, a basic “head to toe” physical examination, written documentation of these findings, and how to orally present these findings. Additionally, we teach them clinical reasoning through encounters with standardized patients who present with specific chief complaints. Clinical skills sessions are taught in small groups of 4 students and led by a clinical faculty member. This component of the course uses mostly standardized patients to provide hands-on practice for the skills being taught. The bi-weekly sessions include clinical skills workshops to teach organ system-specific physical exam techniques in a head-to-toe format. There are also sessions covering communication skills, the approach to challenging patients, counseling sessions for patients with substance abuse, and the use of medical interpreters for non-English speaking patients. The students apply these skills during standardized patient encounters with a variety of chief complaints. This curriculum continues throughout the academic year.

The target audience for this resource was first-year medical students who were learning how to take a history and perform a physical examination. This material could be adapted for use by other medical providers, such as Physician Assistants or Nurse Practitioners. Learners should be familiar with the basics of taking a history, such as using the OLDCARTS mnemonic. An understanding of posterior oropharyngeal anatomy would be helpful but is not required. The ideal context for this resource would be any small group based teaching of physical diagnosis that could be led by an experienced clinician.

We had many sources that we pulled from when developing our module. The information concerning the evidence-based physical examination of a patient with a chief complaint of sore throat was predominately obtained from the JAMA Rational Clinical Examination series [1] and medical textbooks [ 2, 3].

We applied several educational models while to develop our curriculum. Our predominant influence was the Hypothesis Driven Physical Examination [ 4- 7]. The emphasis on tailoring the physical examination to focus on the elements most likely to focus the differential diagnosis was one of our primary goals. Another educational approach used was Gagne’s Conditions of Learning [8]. This theory has been applied to other areas of medical education [ 9, 10].

We utilized a flipped classroom approach [ 11- 14] due to limited class time. The complexities of scheduling numerous standardized patients and desire for very small groups of medical students limited our time in the classroom with students to a few hours every other week. An audio podcast was used as our predominant instructional material, as this media has become increasingly popular in medical education [ 11, 15- 17]. We also included a link an internally developed interactive PDF covering the anatomy of the HEENT examination.

## Methods

We recognized difficulties amongst many of our students in applying clinical reasoning during standardized patient encounters. Course faculty noticed that students were not able to effectively discuss their medical decision making with preceptors after completing a history and physical exam. Students demonstrated effective physical exam technique and obtained adequate histories for their level of training, but they were unable to define why certain examinations must be done or why it was crucial to ask specific questions. We also wanted students to be exposed to the nuances of how practice setting, patient population, and medical specialty might lead to differences in patient management of the same complaint.

There were several challenges that had to be addressed. There was a limited amount of classroom time available for physical diagnosis instruction per week. To keep teaching groups small, a very large number of preceptors from numerous medical specialties was required. Any educational materials would have to be useful to both general practitioners as well as subspecialists that may only infrequently encounter the complaint being discussed. Finally, we wanted our curriculum to be engaging and enjoyable enough for students to be receptive to it.

We started by reviewing the existing script for our standardized patient encounter. We updated the encounter with the patient modeling a case of viral pharyngitis. The students were expected to obtain a history and complete a focused physical exam in 30 minutes. Extra details were removed from the encounter to ensure sufficient time for students to complete the encounter.

The next step was creating a topical outline for the podcast (Appendix 1). This outline was generated using the paradigm of the Hypothesis Driven Physical Exam, and based on previously created script. Emphasis was placed on “red flags” in the history and concerning physical examination findings. The outline followed a traditional SOAP format. This outline was used to provide structure for the podcast recording and was also provided to the students through our institution’s Learning Management System, Brightspace by D2L.

The podcast was recorded by 3 clinicians from different specialties. This included the course director (Internal Medicine), a preceptor from Emergency Medicine, and a preceptor from Family Medicine. The podcast was recorded using a Yeti Blue USB Microphone with a pop filter. The audio was captured with Screen Flow (version 5) on a MacBook Pro. This file was then edited in Audacity and subdivided into multiple files. These files corresponded to a topic listed in the outline. Feedback was then obtained from multiple sources, including a fourth-year medical student, a third-year curriculum director, and an educational researcher.

A facilitator’s guide was written to assist in the discussion after the standardized patient encounter. A copy of this guide was uploaded to a folder in our LMS accessible only by faculty for review prior to the session. A paper copy of this was also provided to facilitator’s on the day of the encounter (Appendix 2).

The podcast and outline of the podcast were uploaded to our LMS one month prior to the session. Students were also assigned an online quiz the weekend prior to their session (Appendix 3). This quiz, accessible from our LMS, was configured to close prior to their session. Students were notified of the existence of this material and provided access instructions for it through their institutional email.

We developed a semi-structured survey with a focus on Level 1 of the Kirkpatrick model [18] to assess on how students react to the learning event. The utilization-focused evaluation was focused on delivery and content. A 7-questions pre-session evaluation survey was administered prior to the standardized patient encounter (Appendix 4). This survey assessed student’s experience with the pre-session materials. We asked for feedback on how we can improve the material.

The students then had a standardized patient encounter that applied the skills they’ve learned. Faculty observed the encounter through a video monitoring system. A standardized checklist reviewing critical components of history and physical examination was use. Feedback was given to the students by both the standardized patient as well as the preceptor immediately after the encounter.

Small group sessions including 2-3 preceptors and 8-12 students were then led in a guided, case-based discussion of medical decision making related to the chief complaint of sore throat. This was guided by the facilitator’s guide. Faculty and students then completed post-session evaluation surveys (Appendix 5). The student post-session survey was focused on how the Doctor’s Lounge helped in patient encounter. Preceptors in the post-session survey were asked to evaluate students, as a group, preparedness and participation in clinical reasoning discussion (Appendix 5).

Data from the student pre-session survey, the student post-session survey, and the preceptor survey were collected and entered into Microsoft Excel. Descriptive statistical analysis was then conducted.

This study was conducted as a quality improvement project to evaluate innovative curriculum.

The model for session with specific chief complaint provided below.

**Figure:**
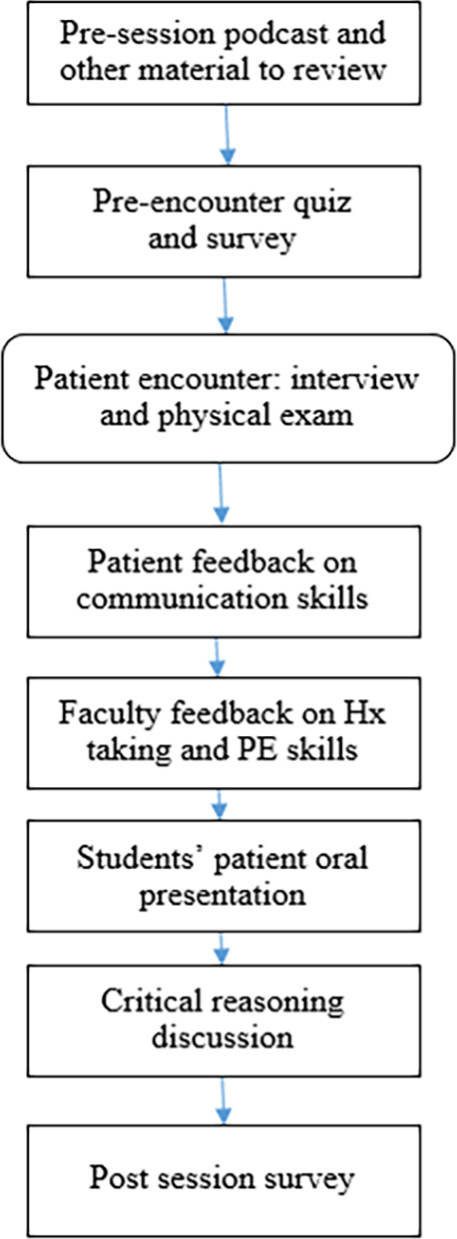


## Results

All 191 students completed the survey with feedback related to the “Doctor’s Lounge”. Only 8 out of 191 students responded they had not used the podcast due to “No time” (N=4), “Can’t access” (N=1), or “Used other resources” (N=3). Preliminary results indicate high utilization of the resource: 52% reviewed all of the podcast, 43% reviewed some of the podcast; 81% reviewed the outline, and 16% reviewed some parts of the outline. More than 45% of the students reported spending longer than one hour using the Doctor’s Lounge podcasts to prepare for class. 54% strongly agreed and 31% agreed that access was easy, 45% strongly agreed and 45% agreed that content was clear, and 46% strongly agreed and 45% agreed that content was helpful for session preparation.

After the session, 37% of students strongly agreed and 47% agreed that Doctors’ Lounge podcasts helped in the encounter; 44% strongly agreed and 43% agreed that it helped with taking a focused history; 40% strongly agreed and 44% agreed that it helped with the physical exam; and 45% strongly agreed and 41% agreed that it helped with discussion of differential diagnosis. Qualitative feedback was analyzed for potential improvement of the resource.

Out of 48 sessions, 39 preceptor surveys were completed and returned. Preceptors strongly agreed (38%) and agreed (46%) that students were prepared for the discussion of the case, and they also strongly agreed (53%) and agreed (29%) that students actively participated in the discussion.

The qualitative feedback received highlighted several areas for improvement. One of the most common requests from students was to shorten the duration of the podcast. We also received requests for different formats of the materials, including different methods of downloading the material and different file types for written materials. Some students also requested more visual aids and suggested the addition of video to the podcast.

## Discussion

The implementation of our educational program was fairly successful. We received positive feedback from faculty members who were experienced with the previous instructional method used for this module. Additionally, upper-level medical students who had experienced the prior method of teaching thought this format of teaching would better prepare students for the clinical wards. Students felt better prepared during the discussion of the medical decision-making surrounding their standardized patient encounter due to use of the podcast and other educational materials provided. Faculty members also noticed satisfactory preparedness of their students prior to the discussion session.

Despite our satisfaction with the improvement that we made to this module, there are several areas that we are actively improving in our second iteration of this module. First, we are introducing additional visual elements to the educational material. This addition will involve the transition of the audio only podcast to a video podcast or “vodcast.” This transition will help clarify visual components of the physical exam and provide a method to demonstrate pathology with pictures of real-life patients. We anticipate that students will have an easier time of grasping concepts such as palatal petechiae when there is an accompanying visual aid. While recording the podcast, it was noted that many of the clinical concepts discussed were very visual in nature.

One of the most consistent themes of the feedback we received from students was that our initial podcast was too long. Our future iteration of this module will have a goal of covering the majority of the same material from the original podcast in less than 30 minutes. Our original podcast was approximately 70 minutes in length, and many students felt this duration was too long to maintain attention. We will preserve the former podcast as a resource for students who are interested in further detail.

The last major change we will be making in our next iteration of this module is the publication of our material in multiple file formats. This publication method will allow for students to use this material on a wider variety of devices. We anticipate publishing our files in MP4 format for video content, MP3 format for an audio-only file, PDF format of the presentation visuals, and an iTunes-compatible AIFF format. This will allow our students to use our materials on a wider variety of devices. By using multiple file formats, this will also allow students to playback our lecture at faster or slower speeds. Our students frequently listen to recorded lectures from their medical school curriculum at 1.5 X speed or double speed.

In addition to improving the quality of our curriculum, we also have plans to expand it into other subject matter. We already have worked on first-year medical student modules addressing the clinical approach to patients with chest pain as well as abdominal pain. We additionally plan to expand this approach to our second-year medical student curriculum. This curriculum is based on a similar model to the first-year medical curriculum and also involves a standardized patient encounter, which is followed by a faculty-led group discussion. These cases, however, are often more complex and will involve more challenging chief complaints better suited to a second-year medical student.

In conclusion, we would strongly encourage faculty at other medical schools to experiment with similar formats to improve their teaching of students on how to properly take a history and perform a physical exam. While there are many aspects that we are actively attempting to improve, the feedback that we received was generally positive. Students arrived better prepared to take full advantage of precious standardized patient time. Their improved performance during group discussions of the medical decision making following the encounter are supportive of our decision to further expand this project.

## Notes On Contributors

Shilpa Brown, MD, FACP, Associate Professor of Medicine, Department of Medicine, Division of General Internal Medicine. She is the Director of the Physical Diagnosis Course and the Director for the Ambulatory Clerkship.

Elena Wood, MD, PhD is an Assistant professor in the Department of Medicine at the Medical College of Georgia. She is the Assistant Director of the Physical Diagnosis Course and the Director for the Standardized Patient program.

Daniel McCollum, MD, Assistant Professor, Department of Emergency Medicine. He is involved in resident education, online education resources, and the FOAMed (Free Open-Access Medical Education) movement. He is the current Assistant Program Director of the Emergency Medicine residency.

Allen Pelletier, MD, FAAFP, Professor of Family Medicine. Dr. Pelletier is involved in patient care and resident and medical student education.

Jennifer Rose, MS, is an Instructional Designer and Systems Analyst who supports Academic Affairs at the Medical College of Georgia. Her expertise is with online learning, and she is actively involved in designing, developing, and maintaining e-learning resources.

Paul Wallach, MD, Professor, Indiana University School of medicine. He is Executive Associate Dean for Educational Affairs and Institutional Improvement.

## Appendices

- Appendix 1 - Outline: https://augustauniversity.box.com/s/44yd5v0t6694n6gb9zx6kzgeuyd889oy
- Appendix 2 - Facilitator Guide: https://augustauniversity.box.com/s/pdan64kmvba0lrgeej7qh8g1josnw9g7
- Appendix 3 - Quiz: https://augustauniversity.box.com/s/hnuqt1hz9rj01l1cpg9sxbyn0c3azzu3
- Appendix 4 - Pre-evaluation Survey: https://augustauniversity.box.com/s/upj4oe9ehxqwcb9enhbs9g8oonh9tykc
- Appendix 5 - Post-evaluation Survey: https://augustauniversity.box.com/s/92p5f4gnh8besfv4sv4nff2one7g94og
