# Supplementary material for: “Doctors’ Lounge” podcast to teach clinical reasoning to first-year medical students
Source: MedEdPublish (2016). 2018 Jun 14;7:132. [Version 1] doi: 10.15694/mep.2018.0000132.1 (PMC10699395; doi:10.15694/mep.2018.0000132.1)
Supplement: Peer review history

# Reviewer report: Reviewer response for version 1

**DOI:** 10.21956/mep.19598.r29105

**Published:** 2018-06-15

**Authors:** P Ravi Shankar

**Affiliations:** 1 American International Medical University

This review has been migrated. The reviewer awarded 4 stars out of 5 Some degree of early clinical exposure is now standard practice in most medical schools. In North America standardized patients play an important role in both learning and assessment of clinical skills. The authors describe a podcast as a method of preparing students for their encounter with the standardized patient so that the limited time available can be utilized more effectively. Doctors’ lounge is a practical and informative title for this series of podcasts. The authors can explain the setting of their medical school, and their class size to provide a background to the readers. Podcasts seem to have been well received by the students. A video podcast will supplement the clinical description and creating a variety of file formats will allow wider use of the material as mentioned by the authors. The authors can briefly explain Gagne’s conditions of learning. This article will be of interest to all curriculum planners and to clinical educators. The increasing adoption of early clinical exposure widens the audience for this manuscript.

Reviewer Expertise:

NA

No decision status is available

# Reviewer report: Reviewer response for version 1

**DOI:** 10.21956/mep.19598.r29104

**Published:** 2018-06-14

**Authors:** Subha Ramani

**Affiliations:** 1 Harvard Medical School, Brigham and Women's Hospital

This review has been migrated. The reviewer awarded 3 stars out of 5 These are important skills to teach novice learners. the authors have designed a useful curriculum to allow students to come in to the clinical rotation more prepared to take a history and perform a physical examination. The paper is well written, well referenced and the appendices give details of the case scripts which can be useful to other educators. In the modern era where time is limited and clinical teachers have multiple competing commitments, such curricula are helpful in preparing students and using clinical teachers' time more effectively.This paper will be useful to clinical educators designing and teaching clinical skills curricula for early medical students.I have a couple of comments. Most curricula need to move beyond Kirkpatrick level 1 on program evaluation. The authors state that they collect quantitative and qualitative data. But brief open-ended comments on questionnaires do not make robust qualitative data. The authors need to interview students and tutors to gather more narrative information with explanations.Many cases need to be developed for this resource to be maximally useful and the authors may be doing this already.

Reviewer Expertise:

NA

No decision status is available
